# Morpho-physiological and biochemical insights into phytoremediation of lithium by sunn hemp (*Crotalaria juncea* L.) and napier grass (*Cenchrus purpureus* Schumach.)

**DOI:** 10.1038/s41598-025-32169-6

**Published:** 2025-12-12

**Authors:** Anushka Alva, HS Likitha Aishwarya, Srivatsa Udupa, Manoj Kumar, Nikhil Kumar Ramesha, Sachin Ashok Thorat, Arya Kaniyassery, Srinivasan Balachandran, Yu-Chung Chiang, Annamalai Muthusamy

**Affiliations:** 1 Department of Plant Sciences, Manipal School of Life Sciences, Manipal Academy of Higher Education, Manipal, 576104 Karnataka India; 2 Bioenergy Laboratory, Department of Environmental Studies, Siksha-Bhavana, Visva-Bharati, Santiniketan, 731235 West Bengal India; 3 Department of Biological Sciences, National Sun Yat-sen University, Kaohsiung, 80424 Taiwan

**Keywords:** Lithium, Metal uptake, Proline accumulation, Physio-biochemical responses, ICP‒MS analysis, Phytoextraction, Plant sciences, Environmental sciences

## Abstract

Phytoremediation is a green and cost-effective strategy used to clean up metal pollutants from contaminated soils. Recently, non-crop species have gained attention for their ability to adapt to and thrive in contaminated environments, making them promising candidates for phytoextraction. However, their effectiveness in accumulating lithium (Li) is not well understood. This study explored the potential of sunn hemp (*Crotalaria juncea* L.) and napier grass (*Cenchrus purpureus* Schumach.) for phytoextraction of lithium a challenging and emerging soil contaminant under controlled greenhouse conditions. Inductively coupled plasma‒mass spectrometry (ICP‒MS) was used to measure uptake of lithium and compartmentalized in plant tissues at different contamination levels. Both sunn hemp and napier grass showed notable capacities for lithium uptake, demonstrating their potential for lithium phytoextraction. The accumulation pattern revealed significantly higher lithium concentrations in roots than in shoots, indicating limited translocation within these plant species. The highest lithium accumulation was recorded in the 1500 µM treatment group in the roots, reaching 7.37 mg/g DW (*p* < 0.001) in sunn hemp and 4.69 mg/g DW (*p* < 0.001) in napier grass. Lithium treatment led to alterations in growth (length and weight of shoots and roots and root architecture), photosynthetic efficiency, gas exchange, and various biochemical traits (total protein and proline contents). Biochemical analysis, including measurements of antioxidant enzyme activities, provided insight into how these plants mitigate lithium-induced oxidative stress. Markedly increased proline levels were detected in both species under lithium stress, suggesting an adaptive, protective mechanism against oxidative damage. The highest proline concentration was recorded in sunn hemp at 1000 µM Li (0.24 mg/g FW) (*p* < 0.01). In this study napier grass exhibited better phytoremediation potential compared to sunn hemp. This comprehensive study highlights the potential of integrating weeds into phytoextraction strategies for the remediation of lithium-contaminated soils. These findings enhance our understanding by use of weeds as effective agents for phytoextraction and sustainable soil remediation techniques and provide crucial insights for the development of weed-based phytoremediation approaches.

**Supplementary Information:**

The online version contains supplementary material available at 10.1038/s41598-025-32169-6.

## Introduction

Metals are naturally occurring intrinsic components of the earth’s lithosphere and play essential roles as indispensable elements and trace constituents^1^. These elements are classified based on their biological significance into essential elements and non-essential elements. Essential elements such as iron, nickel, and manganese, and nonessential elements, such as cadmium, lithium, and lead^1,2^. Metal contamination poses a significant threat to human health and represents one of the most prevalent forms of soil pollution due to the persistent and nonbiodegradable nature. Their long-term retention in soil ecosystems presents considerable risk to the food chain^3^. The migration of pollutants from various sources, including atmospheric and waterborne inputs, into the soil is a major concern, as it can have severe consequences for both human health and food security^4^. Furthermore, metal contamination has both direct and indirect effects on soil microbial communities, thereby influencing mineralization processes and overall soil health^5^. Lithium (Li), a nonessential element, can have detrimental effects on soil properties and enzyme activities, disrupting geochemical cycling, reducing soil fertility, and restricting the availability of essential nutrients to plants. Consequently, Li contamination may result in stunted plant growth, decreased agricultural productivity, it induces physiological stress, affecting photosynthetic pigments, antioxidant systems, and overall decline in plant health and vigour^6^. Metals enter soil ecosystems through both natural and anthropogenic processes. Natural sources include erosion, volcanic eruptions, and sea spray, whereas anthropogenic inputs primarily arise from agrochemical applications, detergent use, improper fuel combustion, mining activities, and industrial emissions^3^. Li contamination is largely attributed to industrial processes, including Li mining, the manufacture of Li-based products, and the improper disposal of lithium-ion batteries (LIBs)^7^.

At low concentrations, Li can stimulate plant growth. The extent of lithium toxicity is influenced by several factors, including its concentration and the tolerance of the treated plant species^8^. While some plants restrict Li uptake beyond threshold levels, others act as hyperaccumulators, storing Li in their tissues^9^. Consequently, effective monitoring and regulation are essential to mitigate Li contamination in the environment.

Traditional remediation techniques for metal-contaminated soils include soil washing, soil replacement, vitrification, thermal treatment, and microbial bioremediation^10^. However, phytoremediation has emerged as a cost-effective and environmentally sustainable alternative, requiring less energy and causing minimal disruption compared to conventional methods^11^. Phytoremediation involves key processes such as metal uptake, transport, compartmentalization, and the activation of tolerance mechanisms. Nevertheless, its practical effectiveness is often constrained by various factors, particularly the need for plant species capable of tolerating high metal concentrations, producing substantial biomass, and remediating soils within an acceptable timeframe to ensure effective metal removal^12^. Among the various phytoremediation strategies, phytoextraction has garnered significant interest. This process involves the uptake of metals from the soil and their subsequent translocation to aerial plant parts, such as shoots and leaves, where they can be harvested and removed from contaminated sites^13–15^. Metal uptake is mediated by specific transporter genes and proteins that influence the efficiency of metal influx^16^. Once absorbed, metals are compartmentalized into different parts to activate tolerance mechanisms to mitigate toxicity and ensure survival^1^.

Li phytoextraction utilizing non-food crops has been the subject of relatively few investigations, underscoring the novelty of this area of study. Because of their high biomass yield, ability to withstand unfavourable soil conditions, and ability to accumulate and remediate a variety of heavy metals, we specifically selected sunn hemp (*Crotalaria juncea*) and napier grass (*Cenchrus purpureus*). Sunn hemp can be used for phytoremediation in contaminated soils because of its strong growth and proven capacity to absorb and withstand heavy metals^17^. Owing to its rapid growth, wide root system, and ability to remove and stabilize a variety of metal pollutants, napier grass is a popular phytoremediation tool^18^. napier grass’s robust tillering, large leaf area, high radiation usage efficiency, deep root system that promotes drought tolerance and nutrient uptake, and effective C4 photosynthetic process are the main reasons for its rapid growth^19^.

The mechanism of Li uptake in plants remains poorly understood. However, it has been proposed that Li transport occurs through multiple pathways involving diverse transporters. Li may enter root cells via either symplastic or apoplastic pathways. If absorbed through the symplastic route, Li is transported through the cytoplasm. In contrast, entry via the apoplastic route may be restricted to the casparian strip, after which Li can still reach the xylem through symplastic transport^20,21^. Non-food crops represent promising candidates for phytoremediation because of their natural ability to thrive in contaminated environments and their high biomass, which can also be used for bioenergy generation^22^. These plants can efficiently store metals in their tissues without experiencing significant adverse effects^23,24^. There are few investigations on plant physiological and biochemical responses to Li stress in natural field circumstances. More molecular, transcriptome, and field-level research is needed to enhance the efficacy of phytoremediation in Li-contaminated soils. In this context, non-food crops and non-food species offer considerable potential for phytoremediation, necessitating further investigations into their uptake, transport, and compartmentalization mechanisms. Such studies will help elucidate the physio-biochemical responses of these plants and enhance their effectiveness in environmental remediation and soil restoration. Phytoremediation research has focused predominantly on conventional heavy metals, while the potential of high-biomass, stress-tolerant non-food crop species for removing emerging contaminants such as Li remains poorly understood. Information on Li uptake, tissue partitioning, and associated physiological and biochemical responses in such species is scarce. Therefore, this study aims to evaluate the phytoextraction potential of sunn hemp and napier grass under varying Li concentrations and reports that both species can tolerate and accumulate Li; exhibit distinct tissue-specific accumulation patterns; and display measurable morphological, physiological, and biochemical responses indicative of their suitability for Li remediation. This study is innovative in analyzing the Li phytoremediation capacity of sunn hemp and napier grass using integrated morphophysiological, biochemical and ICP-MS investigations.

## Materials and methods

### Chemicals and reagents

Acetone (SRL, India), ascorbate (CDH-Central Drug House, India), EDTA (SRL, India), H₂O₂ (Medilise, India), L-methionine (SRL, India), lithium chloride (SRL, India), methanol (Rankem, India), nitric acid (HiMEDIA, India), nitro blue tetrazolium (SRL, India), riboflavin (SRL, India), and Triton X-100 (Fluka Biochemika, Switzerland).

### Assessment of morphological parameters

#### Seed germination

Sunn hemp and napier grass seeds were obtained from Tamil Nadu Agricultural University (TNAU), Tamil Nadu, India. The experiments were performed at Manipal School of Life Sciences, Manipal Academy of Higher Education (MAHE), Manipal, Karnataka, India (13°20’43"N 74°47’18"E). The experiment was conducted from January to October 2024. Uniform and healthy seeds were sown in pots measuring 19 × 17.5 cm with 3.0 kg of soil under greenhouse conditions. Five seeds were sown per pot in the soil, and the germination rate was monitored for seven days post-inoculation (DPI).

#### Plant treatment and assessment of growth characteristics

During the greenhouse experiments, the plants were treated with lithium chloride (LiCl). The seeds were germinated under greenhouse conditions with a photoperiod of 16 h of light and 8 h of darkness at a temperature range of 30–35 °C, 60% of humidity with natural sun light. The 15-day-old plants were treated with three different concentrations of LiCl (500, 1000, and 1500 µM), along with a control (0 µM), every five days for 15 days by incorporating LiCl into half-strength Murashige and Skoog (MS) media. Each treatment consisted of 15 plants (5 plants in each pot with 3 replicates). The plants were harvested after 15 days of treatment. While root length was measured from the base of the stem to the tip of the longest root, shoot length was measured from the base to the apex. Leaf length (from the base of the petiole to the tip) and breadth (at the widest point) were measured with a ruler, and the total number of completely formed leaves was manually counted. A precision weighing balance was used to measure the fresh biomass of the shoots and roots as soon as they were harvested. The samples were oven-dried at 50 °C in a hot-air oven until they reached a consistent weight, after which the dry biomass was measured. After being carefully cleaned to eliminate soil, roots were spread on the transparent plastic film with the help of a needle and then scanned by using HP Jet Scanner. The image formed by the scanner was saved for further analysis. The length of the primary root and the number of lateral roots were measured by using ImageJ Fiji software.

### Assessment of Physio-Biochemical parameters

#### Photosynthetic pigment content

After 15 days of LiCl treatment, mature leaf tissues were collected to determine the chlorophyll and carotenoid contents. A 250 mg sample was homogenized in 5 mL of 80% acetone and centrifuged at 3000 rpm for 10 min at 4 °C. The anthocyanin content (mg/g FW) was measured by homogenizing 250 mg leaf sample in 5 mL of acidified methanol, followed by centrifugation at 4000 rpm for 10 min at 4 °C. The pellets were re-extracted twice, and the resulting supernatant was used for absorbance measurements at specific wavelengths: 663 nm and 645 nm for chlorophyll a and b levels (mg/g FW)^25^, 473 nm for carotenoid levels (mg/g FW)^26^, and 530 nm and 637 nm for anthocyanin (mg/g FW)^27^. The absorbance readings were performed in triplicate via a spectrophotometric microplate reader (TECAN Infinite M200).

#### Measurement of gas exchange parameters

A LI-6400XT gas exchange system (LICOR Inc., Lincoln, Nebraska, USA) was used to assess the net transpiration rate, stomatal conductance, and net photosynthesis rate in fully developed leaves of sunn hemp and napier grass plants treated with different concentrations of LiCl under controlled greenhouse conditions. Data were collected from three plants per treatment, and the net transpiration rate (mmol H₂O m⁻² s⁻¹), stomatal conductance (mol H₂O m⁻² s⁻¹), and net photosynthesis rate (µmol CO₂ m⁻² s⁻¹) were measured for each LiCl concentration^28^.

### Tolerance assessment

#### Protein content estimation 

The 200 mg of leaf tissue collected from the treatment and control plants was homogenized in 2 mL of extraction buffer (0.2 M potassium phosphate buffer supplemented with 0.1 mM EDTA, pH 7.8). The homogenized samples were centrifuged at 15,000 rcf for 20 min at 4 °C. The resulting supernatant was used for estimation of protein content via the Bradford assay^29^. These supernatants were further used to determine the antioxidant enzyme activities.

#### Antioxidant enzyme activity

Enzyme-enriched supernatants were used to assess the antioxidant enzyme activities in Li-treated plants^30^.

i. Superoxide dismutase (SOD) activity was measured by mixing 40 µL diluted (2×) sample with 2 mL reaction mixture containing 50 mM potassium phosphate buffer (pH 7.8), 30 mg/mL L-methionine, 75 µM nitro blue tetrazolium (NBT), and 1% Triton X-100. After the addition of 1 mM riboflavin, the samples were exposed to fluorescent light for 10 min alongside a light blank. The absorbance was measured at 560 nm.
ii. Catalase (CAT) activity was determined by adding 10 µL of crude enzyme extract to 3 mL reaction mixture containing 50 mM potassium phosphate buffer (pH 7.0, diluted 200-fold) and 10 mM H₂O₂. The absorbance was recorded at 240 nm.
iii. Ascorbate peroxidase (APX) activity was measured by adding 10 µL of crude enzyme extract to 1 mL reaction mixture containing 50 mM potassium phosphate buffer (pH 7.0), 0.5 mM ascorbate, and 0.5 mM H₂O₂. The absorbance was recorded at 290 nm^31^.

All the assays were conducted in triplicate. SOD activity (U/g FW) was determined via a pure SOD standard curve, whereas CAT activity (mM/g FW) and APX activity (mM/g FW) were quantified accordingly.

#### Estimation of total proline content

To determine the proline content, fully mature leaves were collected after 15 days of LiCl treatment. A 100 mg leaf sample was homogenized with 3% sulfosalicylic acid (5 µL/mg FW) and centrifuged at 6000 rpm for 5 min at room temperature. The resulting supernatant (100 µL) was mixed with 500 µL of the reaction mixture (comprising 3% sulfosalicylic acid, acidic ninhydrin, and acetic acid in a 1:2:2 ratio). The mixture was heated in a boiling water bath (96 °C) for 30 min and subsequently placed on ice to halt the reaction. One millilitre of toluene was added to the cooled mixture, which was subsequently vortexed for 30 s and incubated for 5 min. The absorbance of the toluene phase was measured at 520 nm via a spectrophotometric microplate reader (TECAN Infinite M200). The proline content (mg/g FW) was quantified by extrapolating values from a standard proline curve^32^.

### Metal uptake and compartmentalization by ICP‒MS analysis

After 15 days of LiCl treatment, the harvested plant samples were separated into roots and shoots, wrapped in aluminum foil, and dried in a hot air oven at 60 °C for 4–5 days. The dried samples were ground into a fine powder using a mortar and pestle. A 50 mg powdered sample was digested in an acid digester with 2% nitric acid until a clear solution was obtained. This solution was then analysed using ICP-MS (Agilent 7480) to determine Li accumulation in different plant tissues (shoots and roots) in triplicate. The calibration was performed using standard solutions at 0, 10, 25, 50 and 100 µg/L, and the data were validated by assessing accuracy, precision and reproducibility.

### Statistical analysis

All the experiments were conducted under greenhouse conditions in biological replicates (*n* = 3) and were repeated twice. The results are presented as the mean values (*n* = 3) with standard deviations. One-way analysis of variance (ANOVA) and Dunnett’s multiple range test were used to differentiate significantly distinct treatment means at *P* < 0.05, and GraphPad Prism 8.0.1 (244) (GraphPad Software, Inc.) was used for statistical analysis.

## Results

### Morphological characteristics

Following LiCl treatments, plants grown under control conditions (0 µM LiCl) and those treated with 500, 1000, and 1500 µM LiCl were monitored for 15 days to assess their growth patterns. The control (0 µM) sunn hemp plants cultivated without lithium treatment presented the greatest growth, followed by those treated with 500, 1000, and 1500 µM LiCl (Fig. S1). In contrast, napier grass displayed the opposite trend, with plant height increasing as the LiCl concentration increased (Fig. S2).

#### Effect of lithium treatments on growth characteristics

**Fig. 1:**
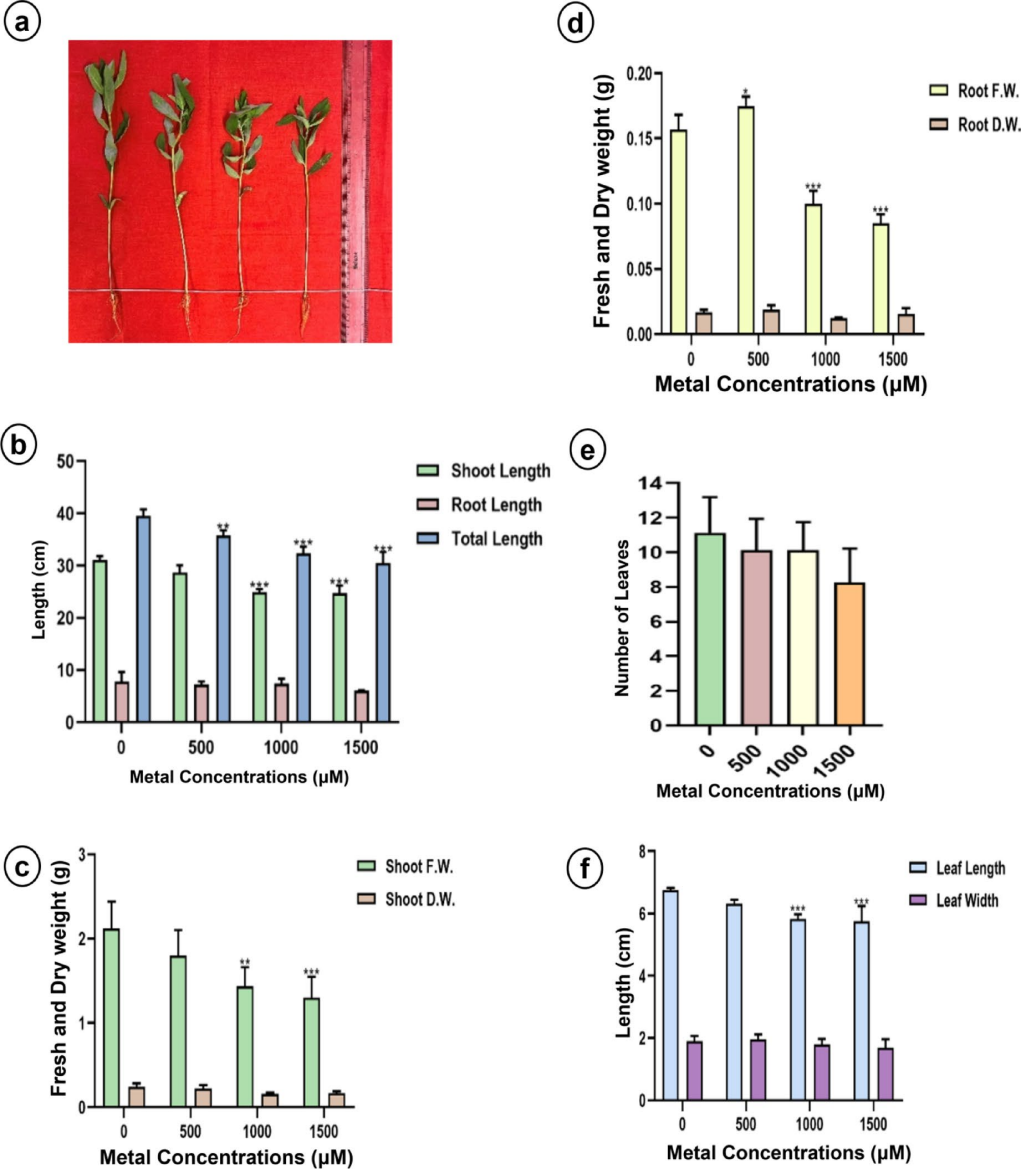
Morphometric characters (**a**) Morphology, (**b**) Shoot, root and total length, (**c**) Shoot fresh weight and dry weight, (**d**) Root fresh weight and dry weight, (**e**) Number of leaves and (**f**) Leaf length and width of sunn hemp under Li treatment. Data are expressed as mean ± SD. Significant differences were determined using ANOVA and Dunnett’s test (*p < 0.05, **p < 0.01, ***p < 0.001).

**Fig. 2:**
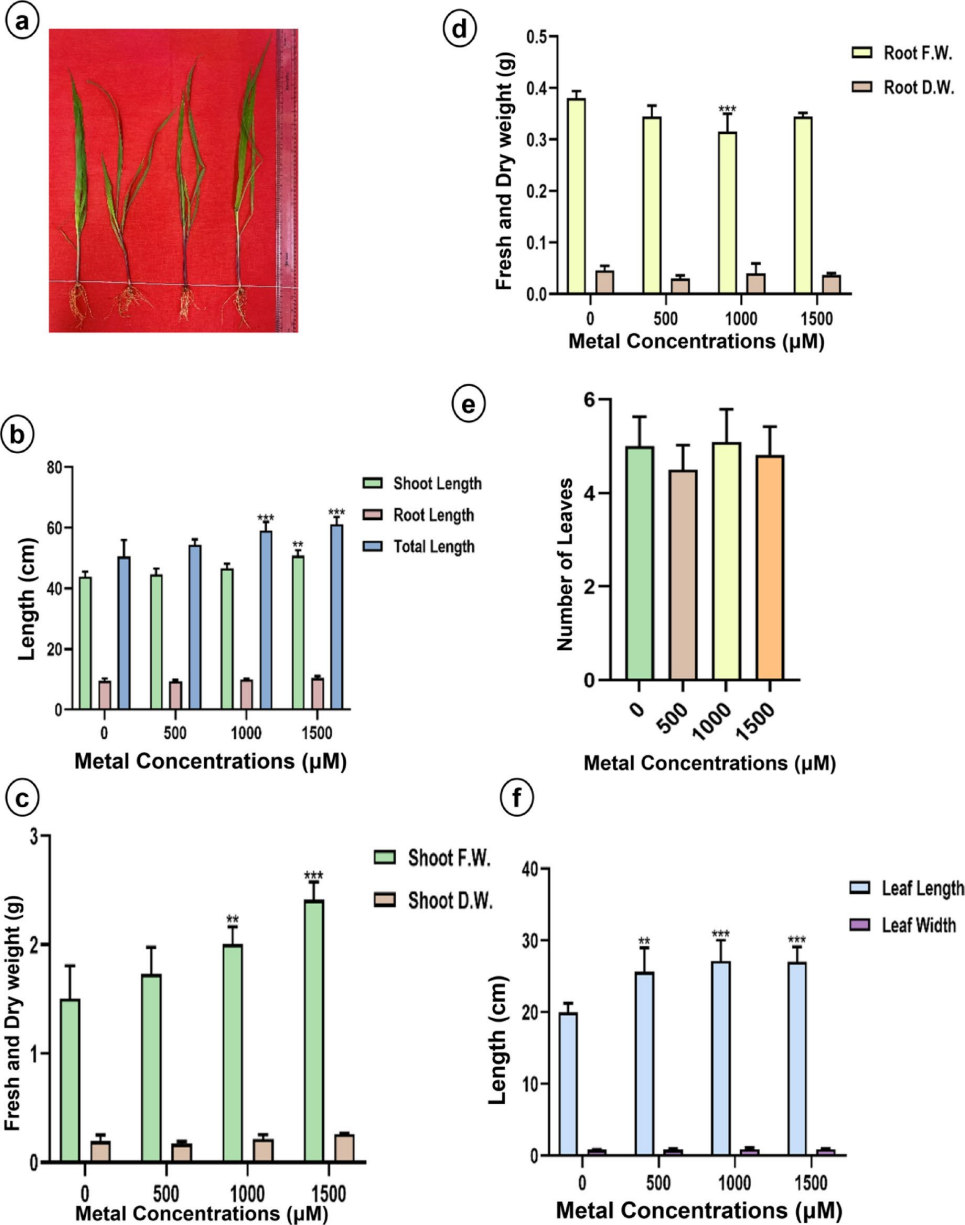
Morphometric characters (**a**) Morphology, (**b**) Shoot, root and total length, (**c**) Shoot fresh weight and dry weight, (d) Root fresh weight and dry weight, (**e**) Number of leaves and (**f**) Leaf length and width of napier grass under Li treatment. Data are expressed as mean ± SD. Significant differences were determined using ANOVA and Dunnett’s test (*p < 0.05, **p < 0.01, ***p < 0.001).

Greenhouse-grown sunn hemp plants were evaluated for changes in plant architecture following a 15-day LiCl treatment (Fig. 1). A reduction in shoot and total length, leaf count, root and shoot fresh biomass, and leaf length was observed in sunn hemp (Fig. 1b, c, d and e, and 1f). Conversely, napier grass exhibited increases in all these morphometric traits, except for fresh root biomass (Fig. 2). Significant differences in total length, shoot fresh biomass, and leaf length were noted between the 1000 and 1500 µM LiCl treatment groups (Fig. 2b and c, and 2e). Although the number of leaves was recorded, no significant differences were detected among the treatment groups for either plants.

#### Effect of lithium treatments on root architecture

**Fig. 3:**
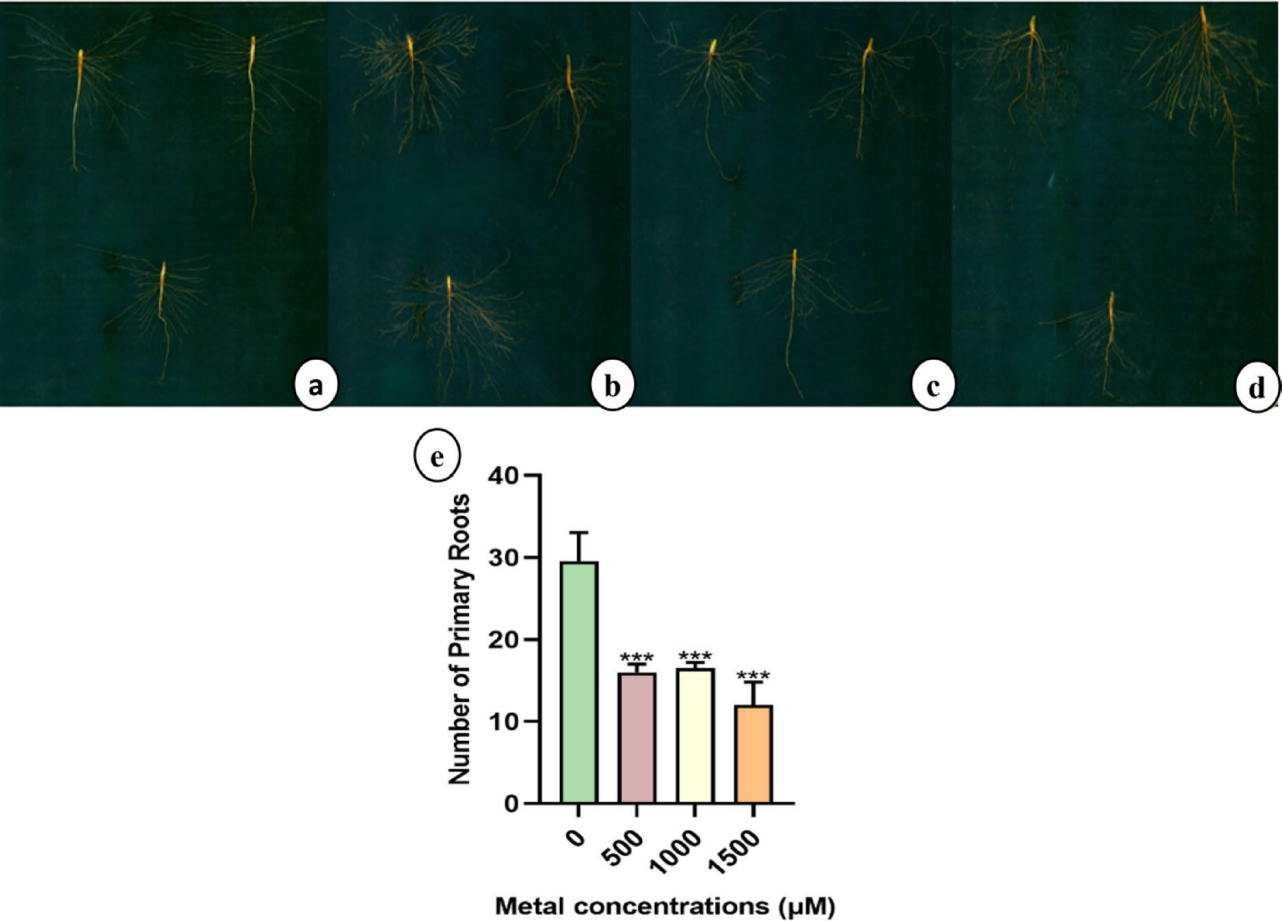
Root architecture and Number of primary roots of sunn hemp (**a**-**e**) and plants treated: (**a**) 0, (**b**) 500, (**c**) 1000, and (**d**) 1500 µM of LiCl.

**Fig. 4:**
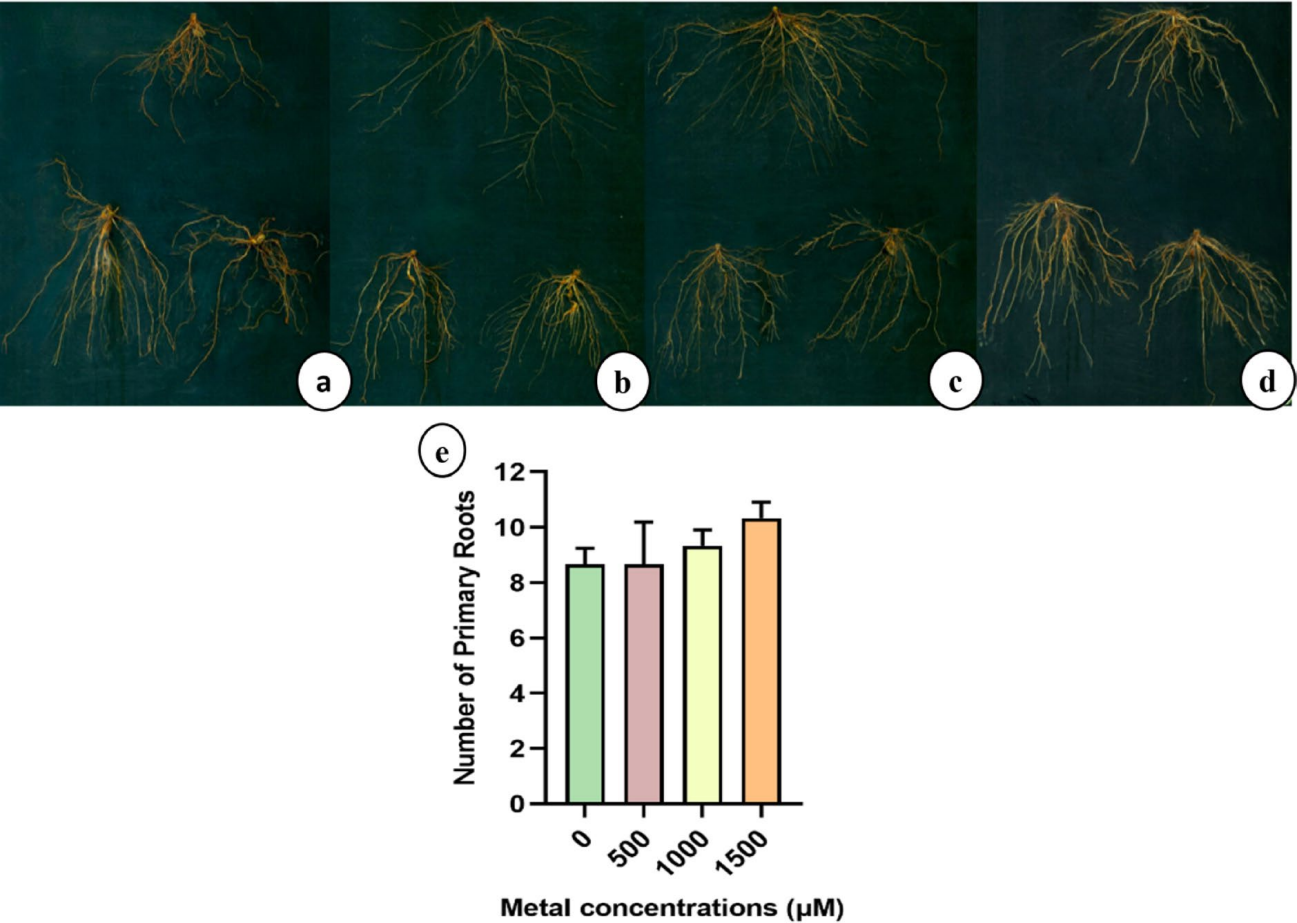
Root architecture and Number of primary roots of napier grass (a-e) plants treated with (**a**) 0, (**b**) 500, (**c**) 1000, and (**d**) 1500 µM of LiCl.

In control sunn hemp the highest number of primary roots was observed with an average of 29 roots (Fig. 3a), whereas all the Li-treated groups showed a significant reduction in root number (Fig. 3b-e). In contrast, napier grass exhibited an opposite trend, with the highest number of primary roots recorded in the 1500 µM treatment group than control (Fig. 4a-e).

### Physio-biochemical parameters

#### Effects of lithium treatments on photosynthetic pigment content

**Fig. 5:**
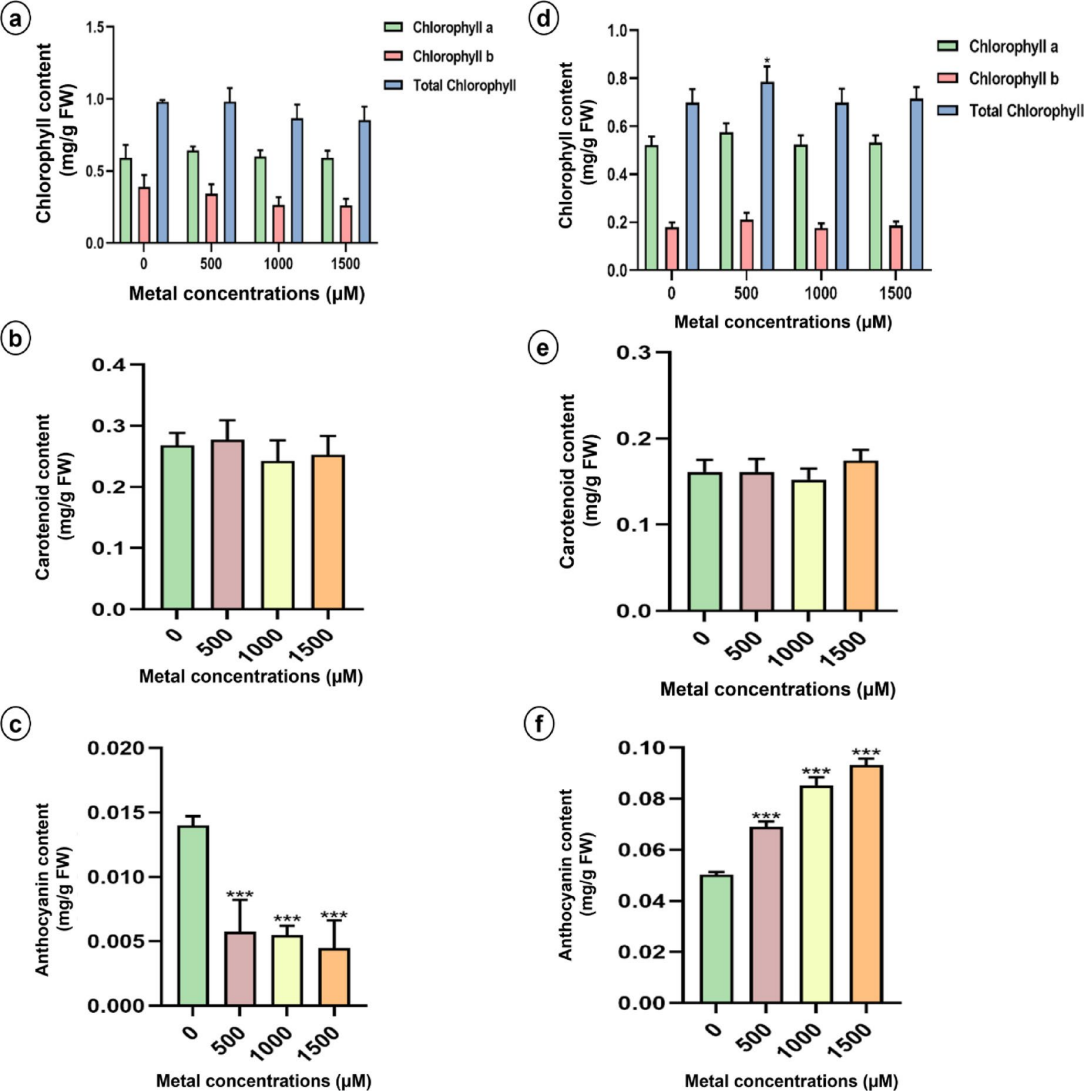
Total chlorophyll content, carotenoid content and anthocyanin content of sunn hemp (**a**-**c**) and napier grass (**d**-**e**) plants grown under Li stress. Data are expressed as mean ± SD. Significant differences were determined using ANOVA and Dunnett’s test (*p < 0.05, **p < 0.01, ***p < 0.001).

Quantitative analysis was conducted to determine whether varying LiCl concentrations affected the levels of photopigments such as chlorophyll a and b, carotenoids, and anthocyanins (Fig. 5). No significant changes were observed in the chlorophyll a and b or carotenoid levels across the treatment groups (Fig. 5a, b, d and e). However, in sunn hemp, the total anthocyanin content was significantly reduced in all the Li-treated groups than in the control group (Fig. 5c). Conversely, in napier grass, anthocyanin levels increased with increasing LiCl concentration (Fig. 5f).

#### Effects of lithium treatments on gas exchange features

**Fig. 6:**
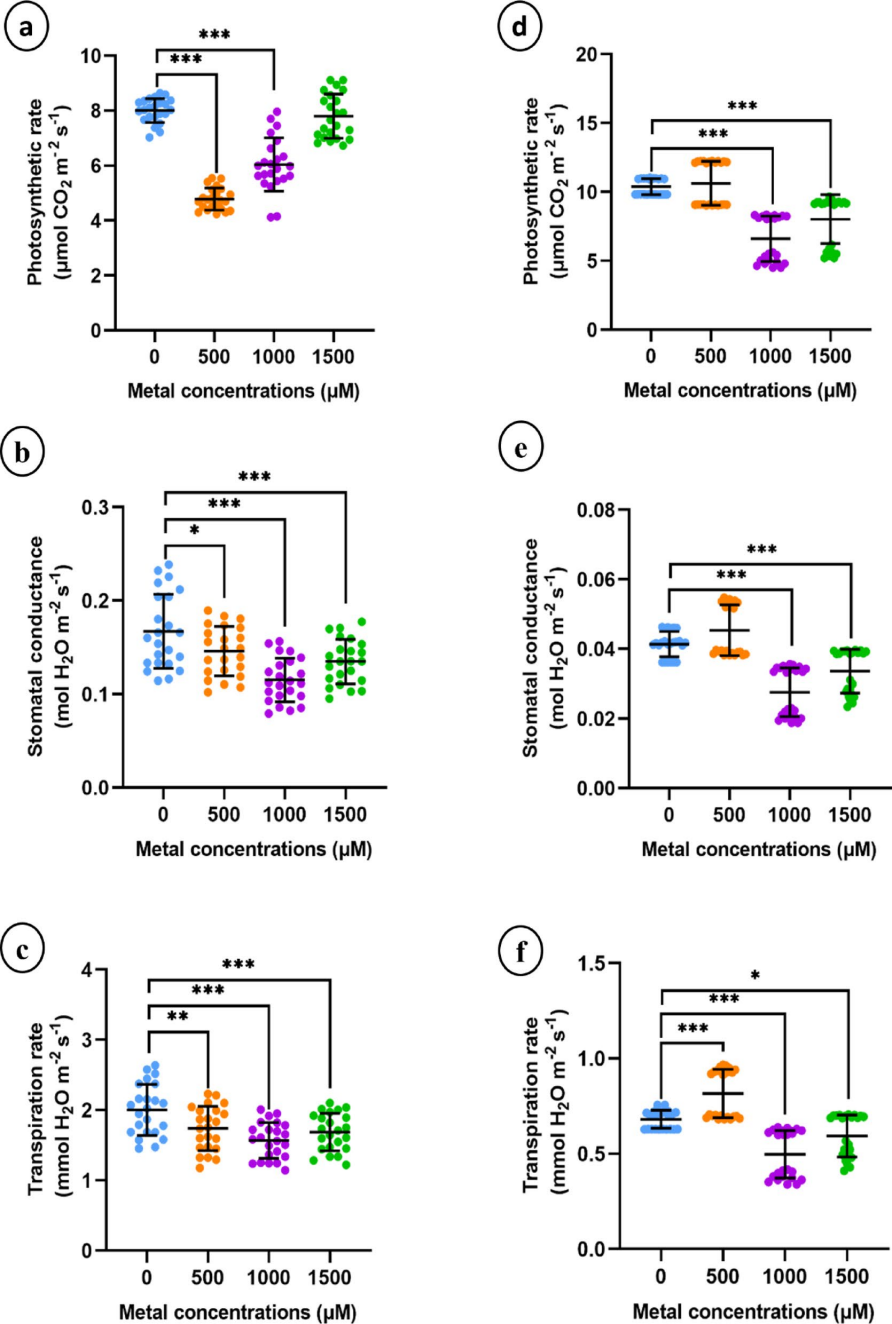
Gas exchange parameters of sunn hemp (**a**-**c**) and napier grass (**d**-**f**) under Li treatment. (a and d) Net photosynthetic rate, (b and e) Stomatal conductance and (c and f) Transpiration rate. Data are expressed as mean ± SD. Significant differences were determined using ANOVA and Dunnett’s test (*p < 0.05, **p < 0.01, ***p < 0.001).

The net transpiration rate, stomatal conductance, and net photosynthesis rate were assessed in leaf tissues after 15 days of LiCl treatment to determine the physiological impact of different LiCl concentrations. The net photosynthetic rate was significantly reduced in sunn hemp at 500 and 1000 µM LiCl and in napier grass at 1000 and 1500 µM LiCl (Fig. 6a and b). Additionally, both the stomatal conductance and transpiration rate decreased significantly in both species, particularly at relatively high LiCl concentrations (1000 and 1500 µM LiCl) (Fig. 6b, c, e and f).

### Effects of lithium treatments on plant tolerance mechanisms

#### Effect of LiCl on plant organic solutes

**Fig. 7:**
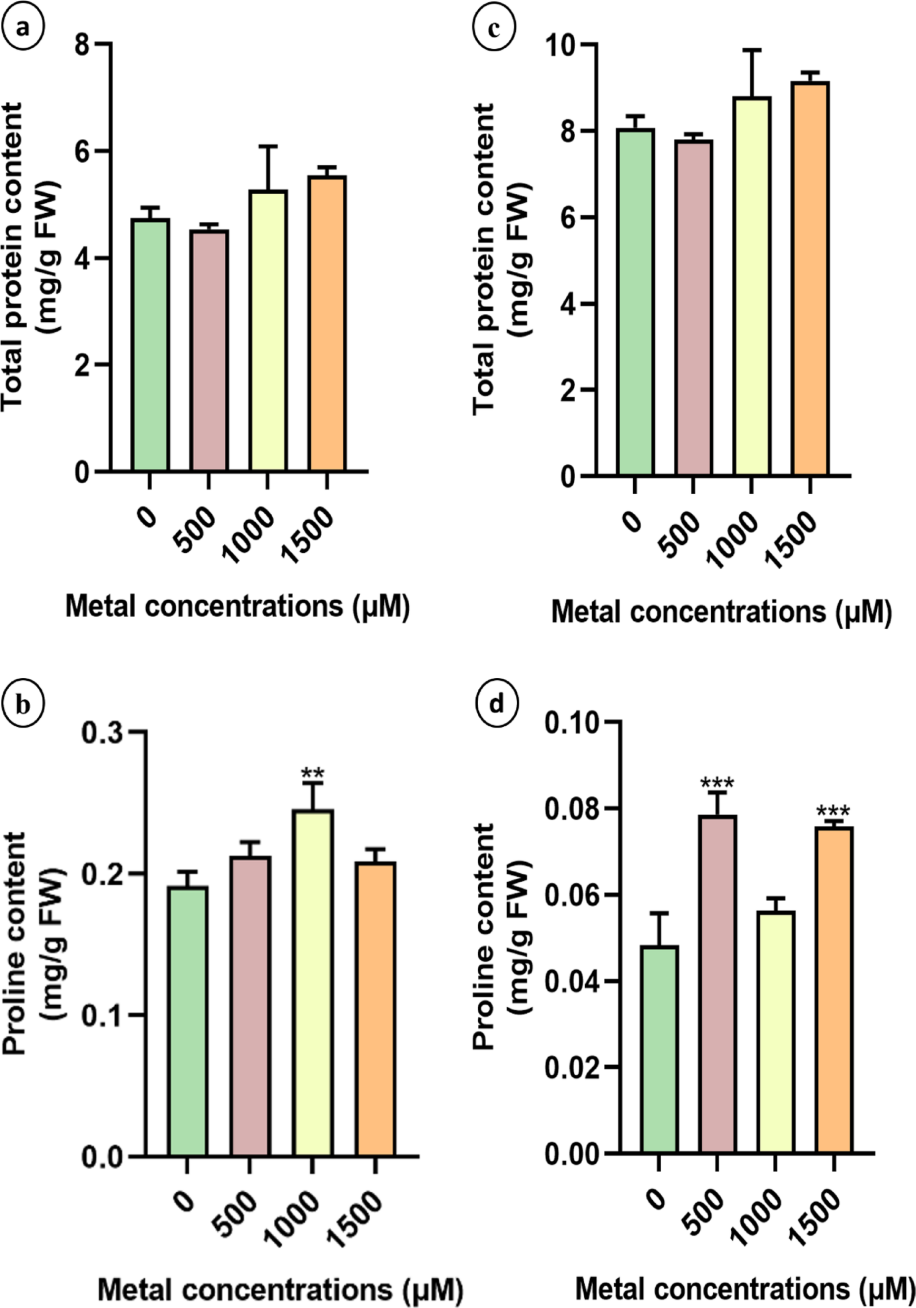
Total protein content and Proline content of sunn hemp (**a**-**b**) and napier grass. (c-d) from leaf tissues during their growth under Li treatment. Data are expressed as mean ± SD. Significant differences were determined using ANOVA and Dunnett’s test (*p < 0.05, **p < 0.01, ***p < 0.001).

There were no significant differences in total leaf protein content observed among the Li-treated groups in either species, despite variations (Fig. 7a and c). However, the proline content increased in all the Li-treated plants. The highest proline concentration in sunn hemp was recorded at 1000 µM LiCl (0.24 mg/g FW), while in napier grass, significantly higher proline levels were detected in the 500 µM (0.079 mg/g FW) and 1500 µM (0.076 mg/g FW) treatment groups (Fig. 7b and d).

#### Effects of LiCl on the antioxidant machinery of plants

**Fig. 8:**
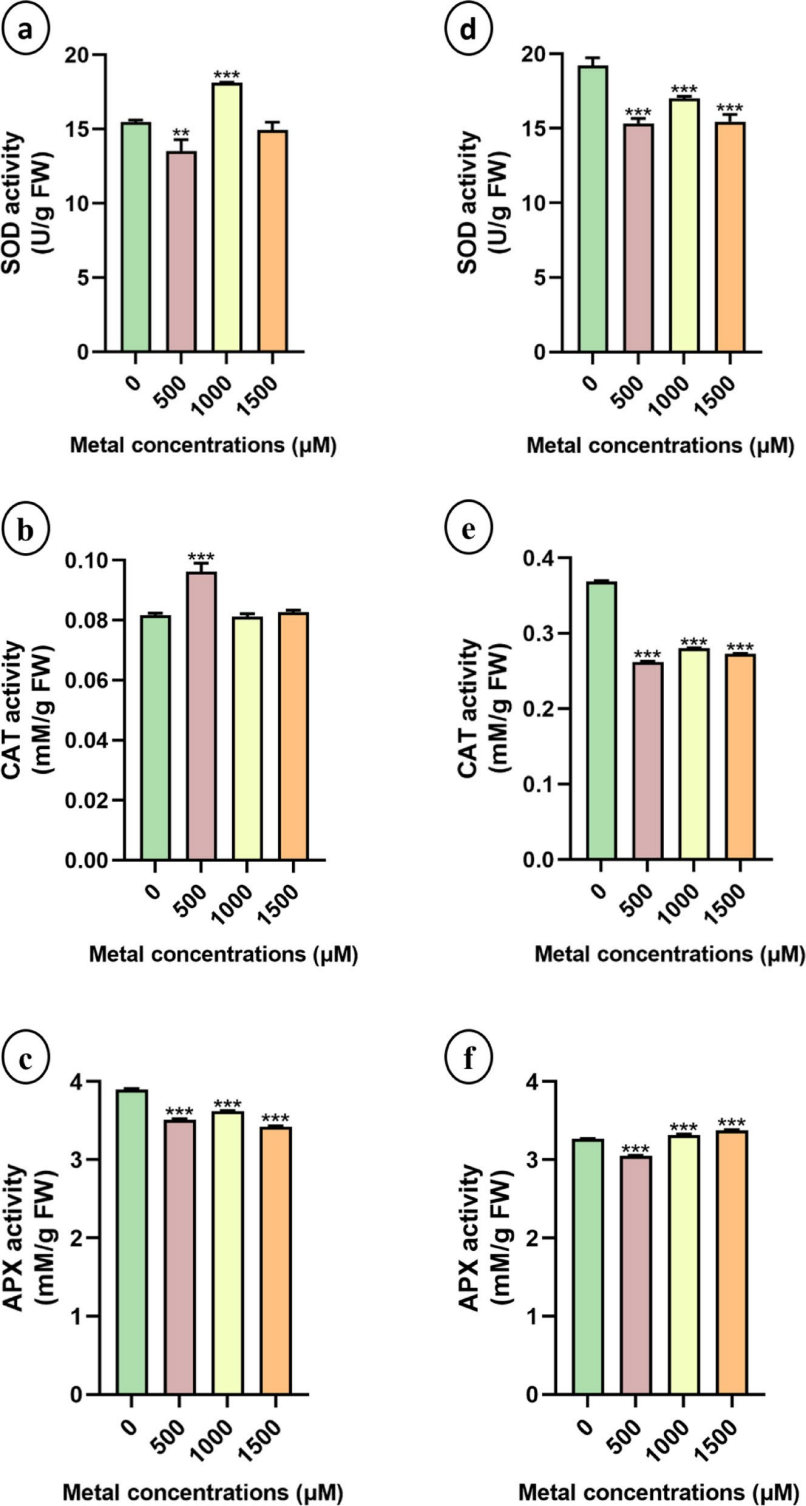
Superoxide dismutase (SOD), Catalase (CAT) and Ascorbate peroxidase (APX) activity from leaf tissues during the growth of sunn hemp (**a**-**c**) and napier grass (d-e) plants under Li treatment. Data are expressed as mean ± SD. Significant differences were determined using ANOVA and Dunnett’s test (*p < 0.05, **p < 0.01, ***p < 0.001).

To assess the impact of LiCl stress, the activities of key antioxidant enzymes involved in reactive oxygen species (ROS) detoxification SOD, CAT, and APX were measured. In sunn hemp, SOD activity significantly increased at 1000 µM Li, whereas the lowest activity was observed at 500 µM LiCl (Fig. 8a and d). No significant changes in CAT activity were detected, except for a notable increase in the 500 µM treatment group (Fig. 8b and e). Conversely, in napier grass, both CAT and SOD activities were significantly reduced in the Li-treated plants. A marked decrease in APX activity was observed in sunn hemp across all the lithium treatments, whereas APX activity increased in napier grass (Fig. 8c and f).

### Uptake and compartmentalization of Li metal

**Fig. 9:**
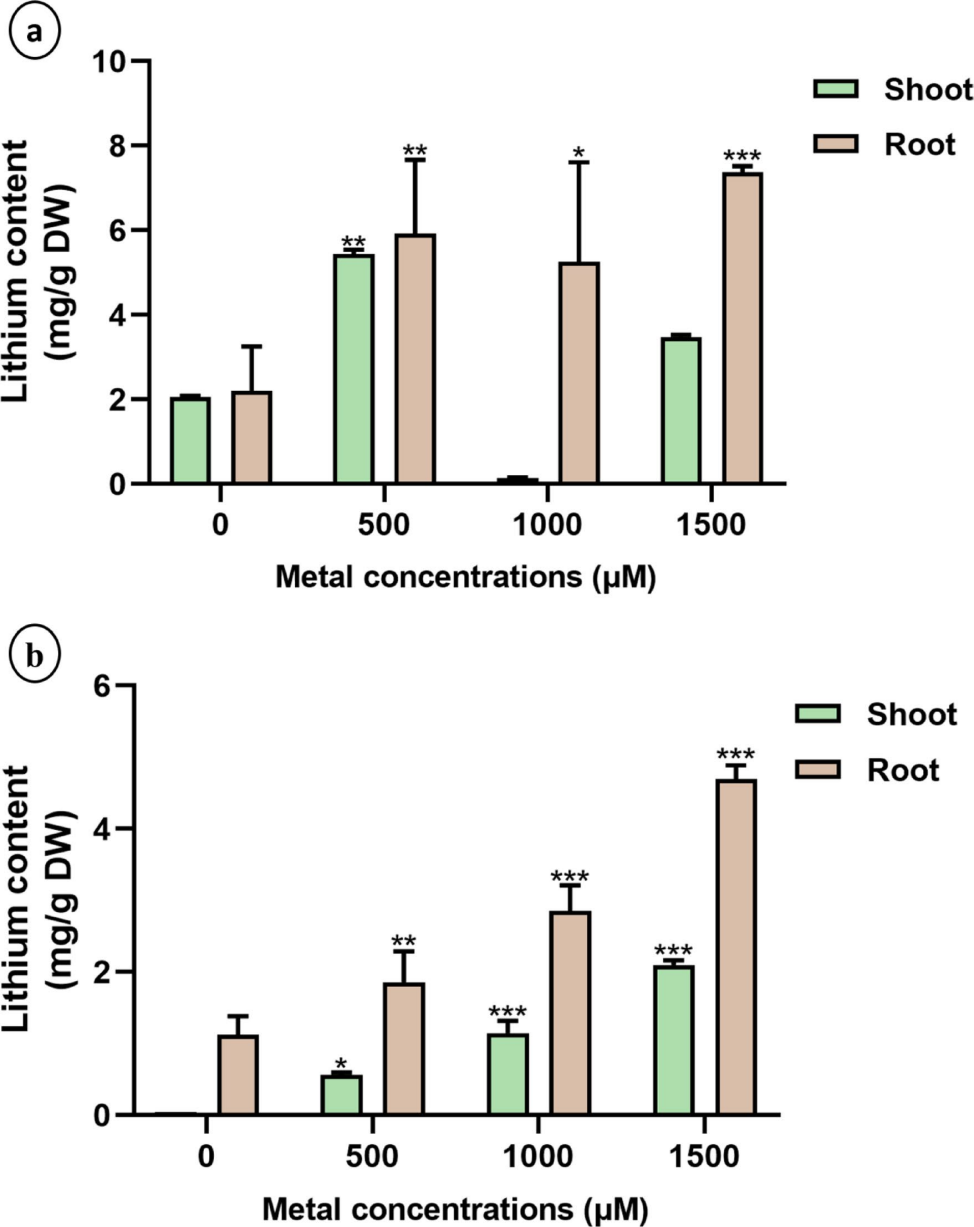
Quantitative analysis of Lithium uptake in shoot and root tissues of (**a**) sunn hemp and (**b**) napier grass plants under Li treatment. Data are expressed as mean ± SD. Significant differences were determined using ANOVA and Dunnett’s test (*p < 0.05, **p < 0.01, ***p < 0.001).

Li accumulation in the root tissues increased significantly (*p* < 0.05) in all the treated plants compared with the control. The highest Li accumulation was recorded in the 1500 µM LiCl treatment group, reaching 7.37 mg/g DW in sunn hemp (3.3-fold increase over control) and 4.69 mg/g DW in napier grass (4.2-fold increase). In sunn hemp shoots, the Li content was significantly higher (*p* < 0.05) at 500 µM (2.1-fold increase), followed by 1500 µM (1.8-fold increase), the control (0 µM), and 1000 µM (Fig. 9a). Physiological regulation and stress responses that limit Li uptake and translocation at higher exposure levels may be the cause of the variation in Li accumulation observed in sunn hemp shoots. In contrast, both the root and shoot Li concentrations in napier grass increased progressively with increasing LiCl treatment (Fig. 9b). Compared with those of the control, the root tissues of sunn hemp and napier grass in the 1500 µM treatment group presented 3.3-fold and 4.2-fold increases in LiCl accumulation, respectively (fold increase was calculated by the ratio of treatment value by that of the control group value).

## Discussion

Metal contamination in soil is one of the most detrimental environmental stressors affecting plants, leading to alterations in their morphological, physiological, and biochemical characteristics^33,34^. Under metal stress conditions, plants typically experience reduced growth and impaired physiological functions^35^. Developmental disruptions also occur due to oxidative stress and the depletion of essential nutrient resources. These nutrients are redirected towards ROS defence mechanisms, ultimately affecting overall plant yield^36^. While the uptake and essentiality of Li in plants remain unclear, previous studies have shown that low Li concentrations can enhance plant structural, physiological, and biochemical features^37^. However, at higher concentrations, Li has been shown to be toxic^38^.

In this study, Li had both positive and negative effects on plant morphology, depending on the species (Fig. 1). Napier grass displayed a significant increase in plant height, leaf length, and biomass (Fig. 2). A similar response has been observed in soybean, where Li treatment at concentrations up to 100 mg/kg led to increased plant height and biomass, which is attributed to a stimulatory response, where subtoxic levels of Li act as mild stressors, stimulating antioxidant activity and enhancing photosynthetic activity^39^. However, under the same Li treatment conditions, sunn hemp exhibited significant reductions in shoot and total length, as well as in leaf length. Furthermore, a substantial decrease in fresh root and shoot biomass was observed, suggesting that sunn hemp is sensitive to Li toxicity at 500–1000 µM LiCl. Li treatment induced dose- and species-dependent responses in sunflower and maize, causing growth inhibition, lipid peroxidation and pigment loss in maize and necrosis in sunflower at relatively high Li concentrations, whereas at relatively low concentrations, it stimulated maize growth^40^. Certain plant species, such as *Brassica juncea*, have shown the ability to hyperaccumulate Li by efficiently absorbing and concentrating it in their above-ground tissues, making them interesting candidates for phytoremediation of Li-contaminated soils^37^. A study reported that exposure of *Hibiscus cannabinus* L. to elevated copper (Cu) concentrations significantly reduced plant growth, biomass and photosynthetic efficiency. Furthermore, Cu toxicity induced oxidative stress, as evidenced by increased levels of malondialdehyde, hydrogen peroxide and electrolyte leakage, suggesting membrane damage. Thus, these findings indicate that metal stress can impair plant growth and physiology through oxidative stress and demonstrate species-specific stimulatory effects^41^.

In this study, significant reductions in stomatal conductance, the photosynthetic rate, and the transpiration rate were observed in response to LiCl treatment (Fig. 6). Similar results have been reported in *Apocynum venetum*, where gas exchange parameters decreased with increasing Li concentrations in the soil^42^. However, chlorophyll and carotenoid levels were not significantly affected by Li application (Fig. 5), a finding that is consistent with a study on sunflower, where no changes in photosynthetic pigments were observed in response to 50 mg/L LiCl treatment^40^. Similarly, spinach treated with Li at concentrations less than 80 mg/kg presented no significant alterations in carotenoid levels^43^.

In contrast, the anthocyanin content exhibited a dose-dependent response to Li stress. In sunn hemp, anthocyanin levels decreased with increasing LiCl concentration, with the greatest reduction observed at 1500 µM LiCl (Fig. 5c). Similar reductions in anthocyanin pigments under metal stress have been documented in *Atriplex hortensis* exposed to metal-contaminated soils^44^. However, napier grass exhibited a dose-dependent increase in the anthocyanin content (Fig. 5f). These findings suggest that anthocyanins may play a protective role in napier grass, contributing to its increased tolerance to Li-induced stress.

Proline, a nonenzymatic antioxidant, plays a crucial role in mitigating oxidative stress by scavenging free radicals generated under metal stress conditions^45^. In this study, proline levels increased significantly in Li-treated plants (Fig. 7b and d), which is consistent with earlier findings that the increase in proline content at higher metal concentrations suggests an enhanced protective mechanism to counter cellular dehydration and oxidative damage^46^. Antioxidant activity is essential for plant survival under metal stress, as plants with weak antioxidant defence mechanisms are more susceptible to oxidative damage^47^. Our results revealed a general reduction in antioxidant enzyme activities in Li-treated plants compared with those in untreated controls, except for APX activity in napier grass, which was elevated under Li treatment. These findings suggest that napier grass may employ a more efficient antioxidant response to mitigate Li-induced oxidative stress (Fig. 8). Similar results were observed upon Cu toxicity in *H. cannabinus*, demonstrating an initial increase in enzyme activity (SOD, CAT and APX), suggesting an adaptive defence response^41^. Because they scavenge ROS, APX and SOD are essential for Li stress tolerance in both monocotyledonous and dicotyledonous plants. Together, APX and SOD shield plants from oxidative damage caused by Li exposure by detoxifying hydrogen peroxide into water and converting superoxide radicals into less dangerous compounds^48^. Li accumulation was generally greater in root tissues than in shoots across all treatment groups (Fig. 9), which is consistent with findings from studies on sunn hemp exposed to nickel, where metal accumulation was predominantly localized in the roots^49^. Li uptake, accumulation, and transport differ among plant species. There are certain plants that are hyperaccumulators or bioaccumulators of Li, such as plants belonging to Solanaceae and Asteraceae, which are known to be Li accumulators^9,40,50^. In a study it was found that Li is transported through the same pathway as K^+^ ions, facilitating its translocation into aerial parts; thus, it has been reported to accumulate more in leaves than in roots^51,52^. However, absorption and accumulation depend on factors such as the Li concentration, plant species, genotype and root structure, which influence its translocation^53^. Particularly in lettuce at relatively low Li concentrations in nutrient solution (2.5–50 mg Li/dm³), more Li accumulated in its roots than in its shoots. Higher external Li concentrations resulted in an increase in the translocation of Li from roots to shoots^50^.

LI uptake and bioactivity in phytoremediation are highly affected by soil physicochemical variables such as pH, electrical conductivity (EC), organic matter, clay content, and soil texture. Acidic soils enhance lithium solubility and availability, whereas organic matter binds lithium, limiting bioavailability. Clay concentration influences Li retention through cation exchange capability. EC frequently rises with Li concentration, changing soil microorganisms and nutrient cycling, hence influencing absorption. These factors affect Li uptake and residual Li in soil during phytoremediation^22,54^. Li residues in soil vary depending on geology and contamination. To assess pollution and phytoremediation efficacy, soil Li residues are measured using ICP-MS or ICP-OES (Inductively Coupled Plasma-Optical Emission Spectrometry). There are studies the impact of Li dosages, clay content, and pH levels on soil Li residues. Such soil tests are critical for understanding Li bioavailability and phytoremediation results^54,55^. The present study has limitation in the residue of Li in soil during our experiment. Further studies are warranted and the experiments on the uptake, transport and accumulation of Li in leaf, shoot, root and soil samples are in progress.

**Fig. 10:**
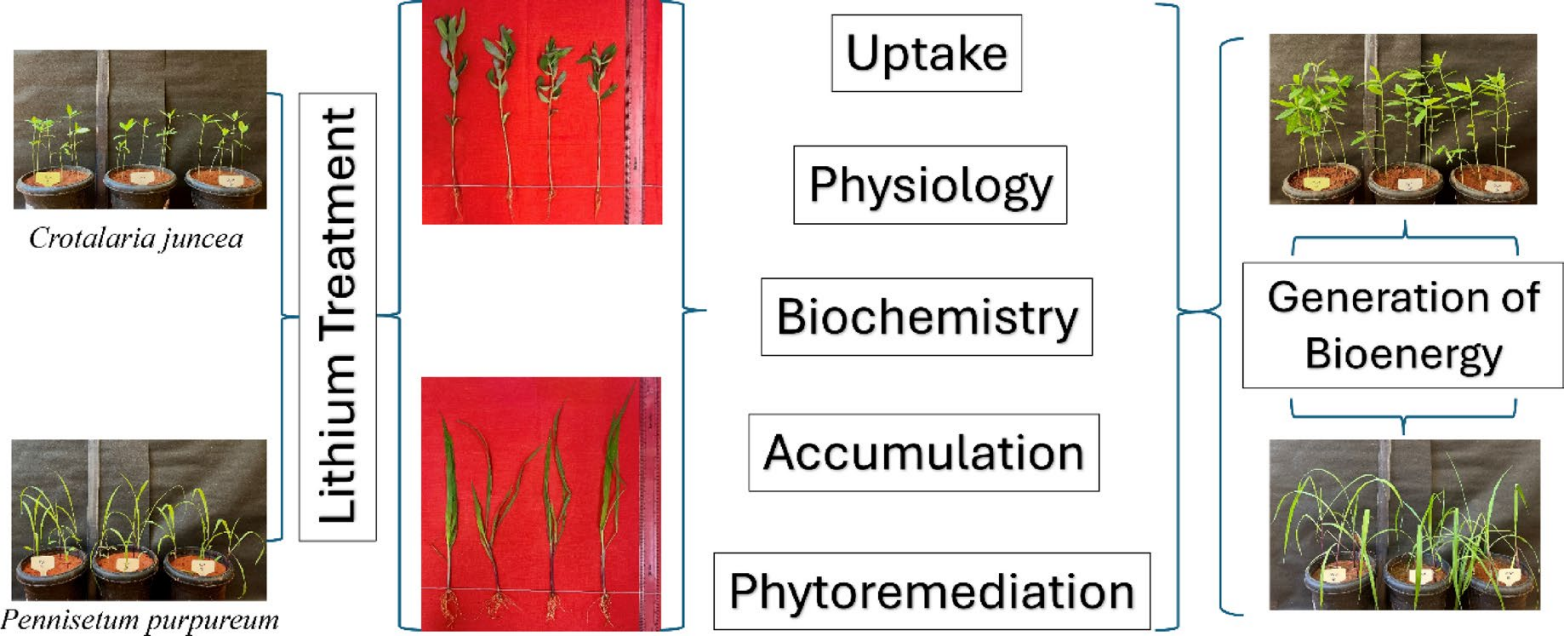
Lithium treatments and responses of sunn hemp and napier grass.

Although Li transport in plants is not fully understood in several transporters, including high-affinity potassium transporters (HKTs), low-affinity cation transporters (LCT1) and nonselective cation channels (NSCC) are known to be involved in Li entry into root cells and accumulation in the cytosol^56^. Furthermore, under high-dose Li treatment, it tends to accumulate in vacuoles and cell walls^57,58^. The brief duration and controlled setting of the Li stress tolerance study hinder its ability to accurately reflect natural field settings. By concentrating primarily on the antioxidant enzymes APX and SOD, other molecular and genetic processes involved in Li tolerance may be overlooked. Furthermore, broader applicability was limited by the lack of thorough investigations of changes in soil types and Li concentrations. The overall findings of the study, uptake, physiology, biochemistry, differential accumulation and phytoremediation are summarized in Fig. 10.

## Conclusion

Sunn hemp and napier grass, both non-food crop species, have demonstrated potential for phytoremediation. This study revealed that Li exposure altered morphometric traits, photosynthetic pigments, biochemical characteristics, metal accumulation patterns, and tolerance mechanisms in sunn hemp and napier grass. While sunn hemp exhibited reduced growth and biomass under Li stress, napier grass presented increased growth and increased shoot biomass production. This comparison study revealed that napier grass outperformed sunn hemp in terms of growth, physiology, and stress tolerance when exposed to Li. Li exposure also led to a decrease in gas exchange parameters and had no significant effect on photosynthetic pigments, except for anthocyanins. ICP‒MS analysis confirmed that Li accumulation was primarily localized in the roots of both plant species, with sunn hemp exhibiting lower translocation efficiency. Additionally, elevated proline levels indicated a protective role in mitigating oxidative stress. These findings suggest that weedy species such as sunn hemp and napier grass may be viable candidates for the remediation of Li-contaminated soils, particularly because increasing Li pollution continues due to the improper disposal of LIB and other anthropogenic sources. However, further research is needed to elucidate Li translocation pathways in plants and to understand the differential responses of plant species to varying Li concentrations. To improve uptake and safe accumulation for phytoremediation, future studies should concentrate on comprehending intricate molecular or transcriptomic analyses to study the mechanisms of Li translocation in plants. Additionally, choosing or enhancing plants for increased efficiency can be aided by researching the physiological and biochemical reactions of different species to different Li concentrations. Furthermore, these contaminated plants can be used for bioenergy generation, which is a sustainable way to handle metal contamination and facilitate the long-term remediation of Li-contaminated soils using non-food species.

## Supplementary Information

Below is the link to the electronic supplementary material.

Supplementary Material 1

## Data Availability

Data generated in this study is provided within the manuscript or supplementary information files.
