# Supplementary material for: Morpho-physiological and biochemical insights into phytoremediation of lithium by sunn hemp (Crotalaria juncea L.) and napier grass (Cenchrus purpureus Schumach.)
Source: Sci Rep. 2025 Dec 12;16:2270. doi: 10.1038/s41598-025-32169-6 (PMC12816059; doi:10.1038/s41598-025-32169-6)
Supplement: Supplementary file 1 — Supplementary Material 1 [file 41598_2025_32169_MOESM1_ESM.docx]

**Supplementary materials**

**Morpho-physiological and Biochemical Insights into Phytoremediation of Lithium by Sunn Hemp (*Crotalaria juncea* L.) and Napier Grass (*Cenchrus purpureus* Schumach.)**

Anushka Alva^1, †^, HS Likitha Aishwarya^1, †^, Srivatsa Udupa^1^, Manoj Kumar^1^, Nikhil Kumar Ramesha^1^, Sachin Ashok Thorat^1^, Arya Kaniyassery^1^, Srinivasan Balachandran^2^, Yu-Chung Chiang^3^ and Annamalai Muthusamy^1,^*

^1^Department of Plant Sciences, Manipal School of Life Sciences, Manipal Academy of Higher Education, Manipal - 576104, Karnataka. India

^2^Bioenergy Laboratory, Department of Environmental Studies, Siksha-Bhavana, Visva-Bharati, Santiniketan - 731235, West Bengal. India

^3^Department of Biological Sciences, National Sun Yat-sen University, Kaohsiung -80424. Taiwan

†Authors contributed equally

***Corresponding Author:**

Dr. Annamalai Muthusamy

Professor

Department of Plant Sciences

Manipal School of Life Sciences

Manipal Academy of Higher Education

Manipal – 576104, Karnataka. India

E-mail: a.msamy@manipal.edu


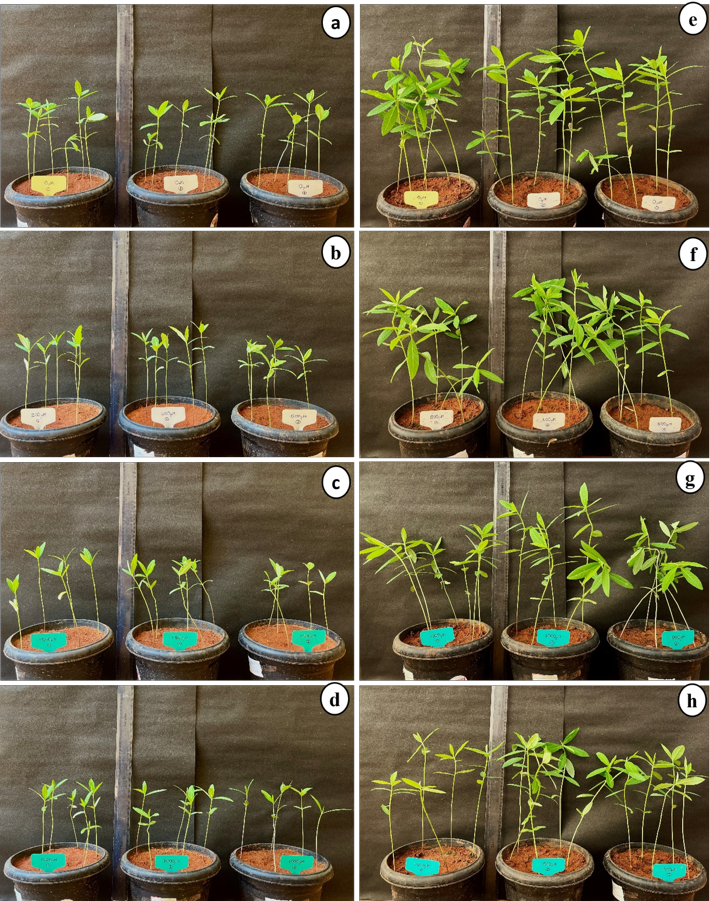


**Figure S1.** Growth of 15 days old (a-d, before treatment) and 30 days old sunn hemp (e-h) under LiCl treatment. (e) 0, (f) 500, (g) 1000 and (h) 1500 μM LiCl.


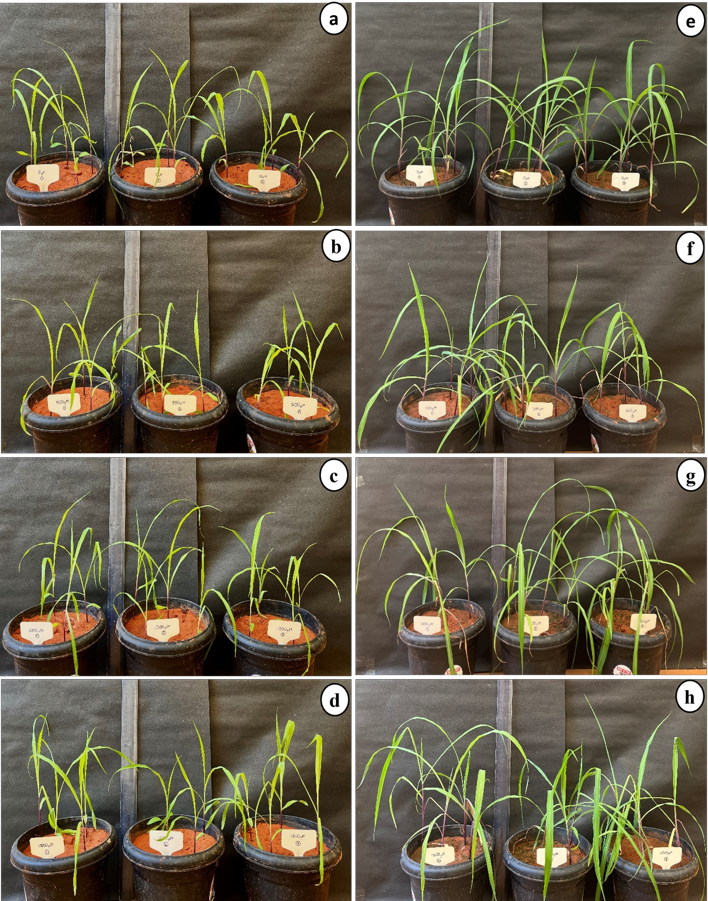


**Figure S2.** Growth of 15 days old (a-d, before treatment) and 30 days old napier grass under LiCl treatment, (e) 0, (f) 500, (g) 1000 and (h) 1500 μM LiCl.
